# Immersive Anatomy Atlas—Empirical Study Investigating the Usability of a Virtual Reality Environment as a Learning Tool for Anatomy

**DOI:** 10.3389/fsurg.2018.00073

**Published:** 2018-11-30

**Authors:** Dirk Weyhe, Verena Uslar, Felix Weyhe, Maximilian Kaluschke, Gabriel Zachmann

**Affiliations:** ^1^Department for Human Medicine, Pius-Hospital, Medical Campus University of Oldenburg, University Hospital for Visceral Surgery, Oldenburg, Germany; ^2^Friedrich – Harkort Schule, Städtisches Gymnasium Herdecke, Dortmund, Germany; ^3^Department of Computer Science, Computer Graphics and Virtual Reality, University of Bremen, Bremen, Germany

**Keywords:** constructivist learning, virtual reality, immersive and interactive anatomy atlas, medical curriculum, virtual dissection

## Abstract

We developed a prototype of a virtual, immersive, and interactive anatomy atlas for surgical anatomical training. The aim of this study was to test the usability of the VR anatomy atlas and to measure differences in knowledge acquirement between an immersive content delivery medium and conventional learning (OB). Twenty-eight students of the 11th grade of two german high schools randomly divided into two groups. One group used conventional anatomy books and charts whereas the other group used the VR Anatomy Atlas to answer nine anatomy questions. Error rate, duration for answering the individual questions, satisfaction with the teaching unit, and existence of a medical career wish were evaluated as a function of the learning method. The error rate was the same for both schools and between both teaching aids (VR: 34.2%; OB: 34.1%). The answering speed for correctly answered questions in the OB group was approx. twice as high as for the VR group (mean value OB: 98 s, range: 2–410 s; VR: 50 s, 1–290 s). There was a significant difference between the students of the two schools based on a longer processing time in the OB condition in School B (mean OB in School A: 158 s; OB in School B: 77 s). The subjective survey on the learning methods showed a significantly better satisfaction for VR (*p* = 0.012). Medical career aspirations have been strengthened with VR, while interest of the OB group in such a career tended to decline. The immersive anatomy atlas helped to actively and intuitively perform targeted actions that led to correct answers in a shorter amount of time, even without prior knowledge of VR and anatomy. With the OB method, orientation difficulties and/or the technical effort in the handling of the topographical anatomy atlas seem to lead to a significantly longer response time, especially if the students are not specially trained in literature research in books or texts. This seems to indicate that the VR environment in the sense of constructivist learning might be a more intuitive and effective way to acquire knowledge than from books.

## Introduction

Using digital media for learning purposes is a much more discussed field of research than one might suspect. There were already theoretical considerations in the 80s to use computer games in class (1–3). The development is driven by the hope to learn more easily and effectively. The growing interest of researchers, educators, parents, players, and game developers has led to the development of so-called “serious games” and thus prepared the ground for digital game-based learning. The serious game research began in the early 90s. The number of publications has increased exponentially since then and is currently in a consolidation phase (4). The fields of application of serious games are manifold and they are already used in the military or for further training in companies (5). The discussion of the use of serious games in school has become increasingly intense in recent years because a playful environment is assumed to have a higher motivation potential for learning (6–11). Nowadays, especially the use of Virtual Reality (VR) serious games in school and higher education is discussed intensively, especially with regard to the intrinsic motivation potential (12, 13). In addition, a meta-analysis shows a high learning efficiency with VR in higher education (14).

**Figure 1:**
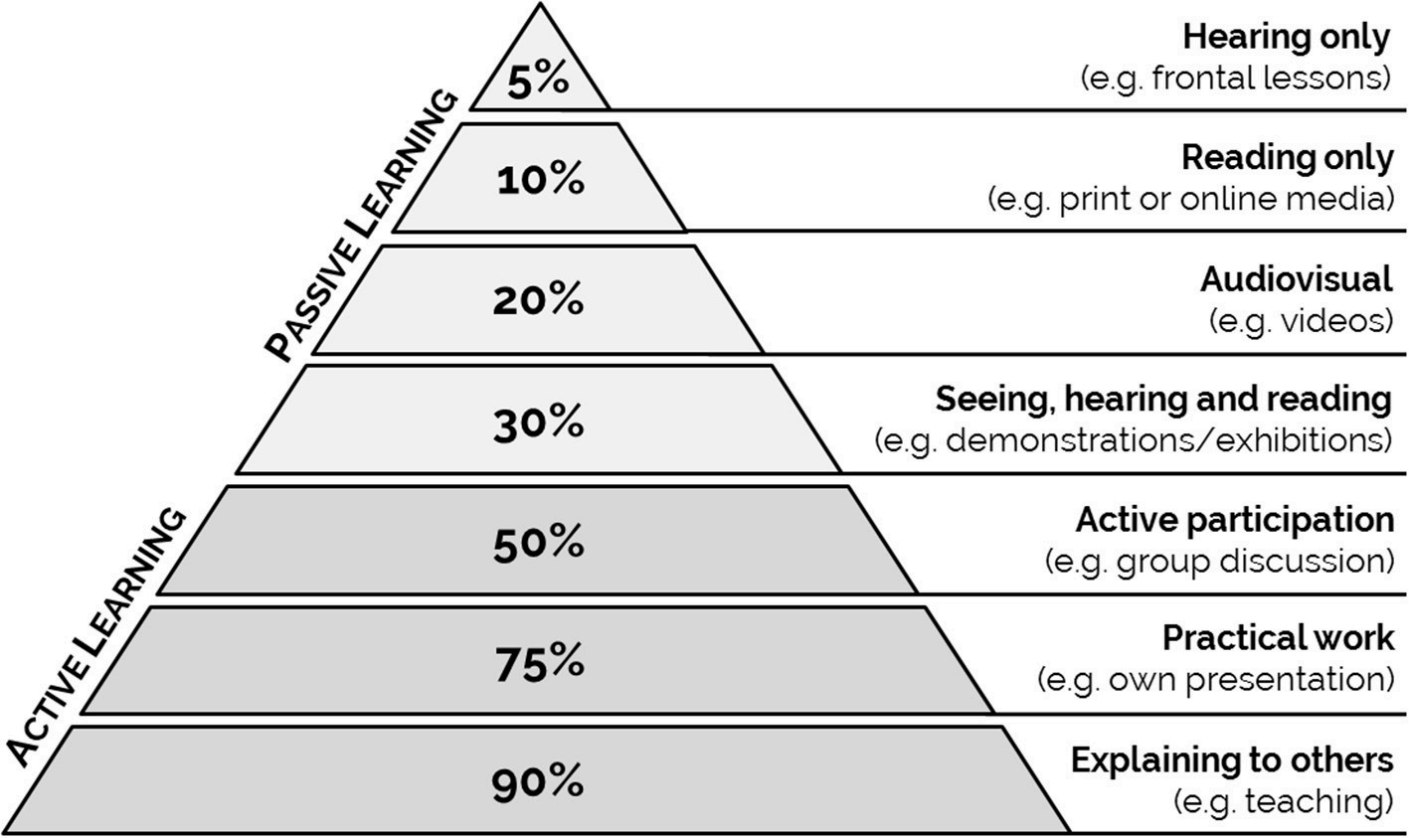
The amount of knowledge that can be retrieved depends on the teaching method. Learning pyramid based on the learning pyramid published by the NTL Institute for Applied Behavioral Science.

This could for instance be explained by the constructivist view of learning, which claims that human experience and learning are subject to certain construction processes (15–18). Learning is influenced by sensory, neuronal, cognitive, and social processes. Neubert et al.'s approach claims that every learner learns on the basis of his own “experience,” and additionally adds his own values, beliefs, patterns, and previous experiences to the new information. On the basis of the learning pyramid, which is established in the community advocating constructivist learning, it is therefore assumed that only an average of 10% of what is read is remembered, since reading is a passive learning process. Practical actions, on the other hand, are active learning processes and already lead to a correct reproduction of what has been learned in 75% of all cases (Figure 1).

Against the background of practical actions, virtual worlds open up new possibilities to support learning processes more strongly through active interactions such as moving things, acting and being able to more strongly involve in the subject matter. Through this visual exploration and the virtual touching of objects, and the associated high immersion, learning content seems to be conveyed more intensively. Based on the constructivist learning theory, a higher learning efficiency is conceivable through these mechanisms.

So far, little is known about the application of VR technology in a medical learning environment (19–21). For instance, positional relationships in anatomy are difficult to convey by means of books (22). Here, VR might probably unfold the existing potential of the three-dimensional representation. In addition, a large number of examinations have shown that surgical Skills-Lab training improves individual performance and reduces the error rate (23–26). However, it is also known that typical carcass training and/or boxing trainers are either not sufficiently available or are perceived by the residents as unattractive courses (27–29). Therefore, one motivation of our group to develop the immersive and interactive anatomy atlas (which in the future will be developed into an immersive surgical simulator) was the intention to create a learning tool which raises motivation.

We developed a prototype of the immersive anatomy atlas, featuring a virtual operating theater, where anatomical structures and arrangements of the human body can be explored through an immersive dissection. With this pilot project, we examined the feasibility and usability of the immersive anatomy atlas in comparison to the open book method (OB) under exam conditions in 11th grade students from two different high schools randomized into two groups (VR vs. OB). As a measure for the usability and ease of handling of both learning tools, we determined the error rate for 10 questions posed to each student, in addition to the duration for answering correctly.

## Materials and methods

### Immersive anatomy atlas

**Figure 2:**
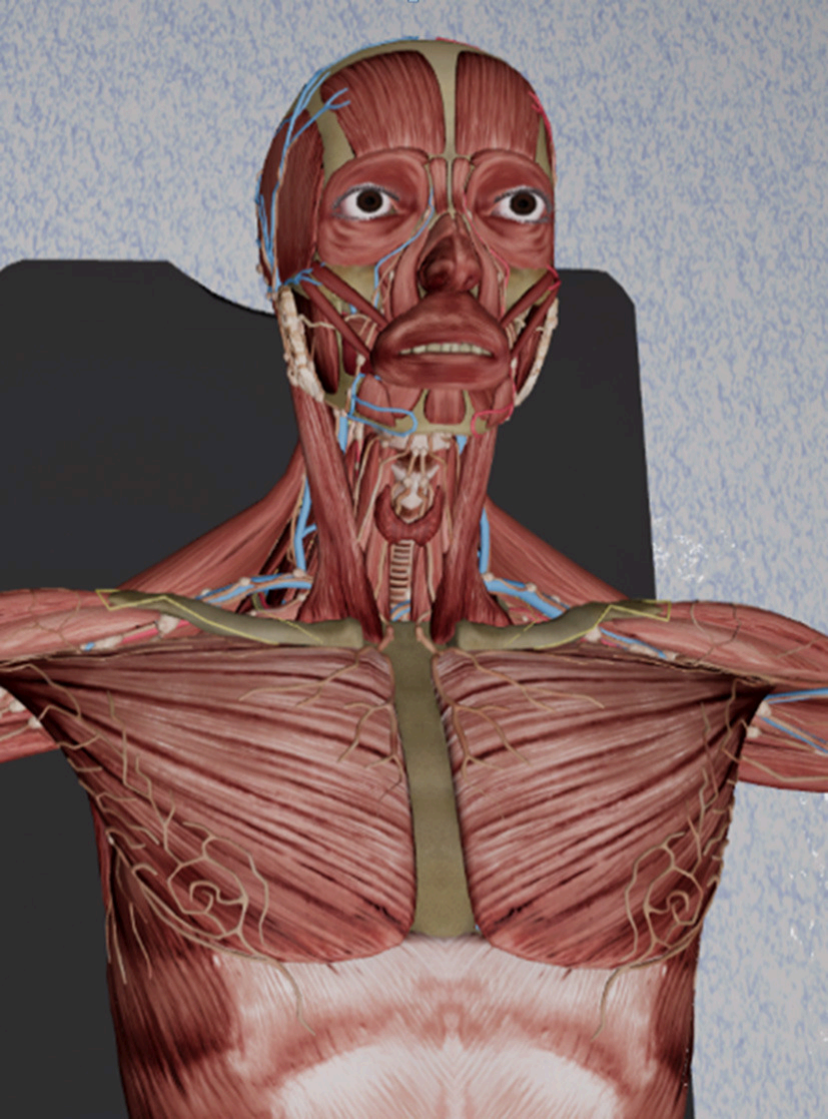
A screenshot of our immersive anatomy atlas, showing a detailed human anatomy model.

By wearing a headset with integrated screens for each eye, special lenses and software to bend the image, the all-round view of a virtual reality is simulated. The user is placed in a virtual operating room with realistic lighting and medical equipment. A virtual dummy with precise human anatomy is placed on the operating table, ready to be inspected (Figure 2). Individual organs can be manipulated via bi-manual controllers. The virtual hand is closed by pressing the action button on the back of the right controller with the index finger. For anatomical structures that are currently held in the hand, further information can be called up. The left controller can be used to hide nearby anatomical structures. Each organ will snap back to its original pose when it is within a translational and rotational threshold of said pose. Additionally, a context menu allows switching the controller-assigned actions, for left-handed people, as well as resetting the whole scene, including organ arrangement.

The grabbing action uses a specially designed collision detection algorithm that only allows grabbing of structures that are reachable from the outside. This increases the intuitiveness when interacting with the virtual environment. Collision detection is performed on the raw mesh structure, instead of being approximated by bounding volumes.

A video of the version of the anatomy atlas used in this study can be viewed here (Supplementary Video). There is also a video of the latest version, which has some additional features for manipulating the anatomical dummy (https://youtu.be/JY50Wjh-olw).

### Learning environment in the two high schools

We conducted our study in collaboration with two high schools (“school A” and “school B”). We chose those two schools in order to draw a larger number of participants. Also, these two schools follow different teaching approaches, which allowed us to consider the effect of an immersive teaching method within different teaching contexts.

The eleventh-graders of the two high schools are normally introduced to new respective topics by different learning methods. In high school A, students typically receive what is known as “smart” teaching geared toward utilization of digital media. Lectures by students are regularly delivered as power-point lectures, which are then shared at school via smartphone and projector. The communication during the lessons is also carried out via the school's own internet-based communication platform. In addition, modern “whiteboards” and tablet PCs are permanently used as interactive teaching media in the classroom. In high school B, students are explicitly trained in research in books, texts, and online media. Visual media are used more cautiously in high school B, but experimental investigations and a dialogue-based transfer of knowledge are promoted.

Medical students show very differing levels of anatomical knowledge, whereas the high school students are more comparable since they had no specific knowledge of anatomy. Therefore, we conducted the experiment with high school students to avoid bias due to heterogenous knowledge.

### Study design

After the development of the immersive anatomy atlas, the ease of use of the atlas was tested via an exam with 10 questions at two different high schools in comparison to an exam in open book format. To test the usability, the questions were formulated from the perspective of a high school student of the same age from high school A in the context of a school research project, to ensure understandability and appropriateness of the questions. The questionnaire consisted of three multiple choice questions and six questions with freely formulated answers (see Table 1). In addition, a sketching task had to be completed (question 10). Since comparable tests are not available, this non-validated test was used.

**Table 1:** Translated question catalog and correct answers.

**Question**	**Correct answer**
How many lobes does the right lung have? 2, 5, 6, 3, 4?	3
What is the structure between stomach and lungs look like?	Diaphragm
Name the annular muscle that surrounds the eye	Musculus orbicularis(oculi)
Name the Latin term of the kneecap	Patella
Name the nerve structure connecting the brain to the spinal cord	Medulla or brain stem
How many muscles are in direct contact with the femur? 13, 9, 6, 21, 27	13
How many parts does the calf muscle consist of?	3
Where is the thyroid gland? In front of or behind the windpipe?	In front of
What is the right temporal muscle (in German: “Schläfenmuskel”) called in Latin?	Musculus temporalis
Sketch the Achilles tendon in proportion to the leg	Complete a schematic drawing

Before the students were included in the study, the parents and participating students were informed in writing. Furthermore, a written declaration of consent was obtained for participation in the study, publication and potential photography.

#### High school A

The test was carried out by a high school student of the same age. All participating students were randomized into one group using the immersive anatomy atlas (VR group; *n* = 5) and into another group provided with a topographic anatomy atlas and separate anatomy tables (OB group; *n* = 5) (22, 30). The average age of the participants was 17 years (range: 16–17), the gender distribution was equal. The questions were answered on 2 consecutive days (day 1: VR Group, day 2: OB Group) There was no time limitation. At the beginning of the investigation, the analysis of response times and error rates was pointed out. The participating test persons were not allowed to discuss the contents of the examination.

**Figure 3:**
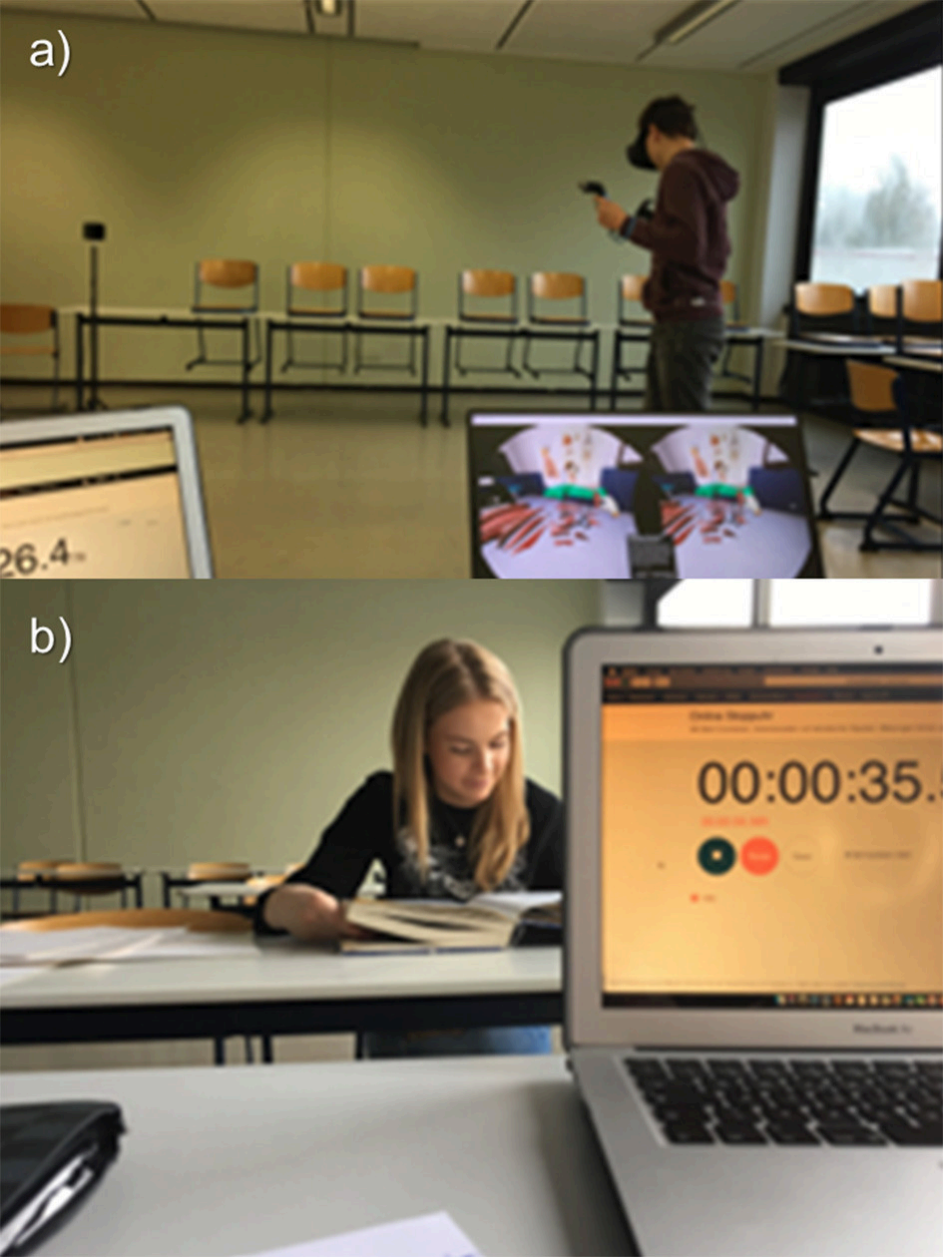
**(A,B)** Photos of the conduction of the study at school A.

At the beginning of the test, the participants of both groups were given an orientation time of 5 min. Within this time frame, the operating instructions of the immersive anatomy atlas and the familiarization in virtual space as well as the review of conventional teaching aids for orientation in the open book test (Figures 3A,B) were given for each group as a whole. The participants in both groups were under constant supervision. The questions were put to both groups orally. During the test, the time required to answer each question was measured and documented by the test manager.

#### High school B

In high school B, the same questions were asked in a group of *n* = 18 students specially trained to understand texts. Students were randomized to each group (OB: *n* = 10; VR: *n* = 8). The teaching aids in the “open book” to compensate for a potential methodological advantage in the VR group (31). In addition, two questions were added to the questionnaire for subjective appraisal of both teaching methods:

***Translation of the questions regarding the subjective appraisal of the teaching*** ***methods:***

Assign a school grade for the teaching unit: (German school grade system: 1–6, 1 being the best grade)

Has this teaching unit given you the idea of taking up a medical profession (doctor, physiotherapist, paramedic, nursing, etc.)?

Yes Maybe No I had this idea before

The trial was supervised by scientific staff of the University Clinic for Visceral Surgery at the Pius Hospital Oldenburg. The test was carried out according to the specifics described above for high school A. The participants were on average 17 years old (range: 16–19 years). The gender distribution was not equal. *N* = 13 girls (OB = 7, VR = 6) and *n* = 5 boys (OB = 3, VR = 2) from high school B took part in the study. An exchange about the content of the questions and the examination situation was impossible both between the pupils and between the schools.

### Statistics

The error rates and the processing time were analyzed. The data was tested for normal distribution using Shapiro-Wilke's test. For the normally distributed error rates, a three-way ANOVA was used to calculate statistical differences. The independent variables used were school affiliation (Gymnasium A and B), teaching conditions (VR and OB) and question number (Q1 to Q10). The processing time data was not distributed normally. Here a Kruskal-Wallis one-way ANOVA was calculated with the four groups VR in school A, VR in school B, OB in school A, and OB in school B. The Mann-Whitney *U-*Test was used to compare the subjective appraisal of the teaching methods. All statistical tests were performed with Sigma-Plot 12.0, the graphics were created with Origin 2016.

## Results

The 28 participants of both high schools were motivated and concentrated. All students conducted the test in a very disciplined manner. All questions were dealt with and in the case of unclear solutions, especially in the OB group, the answers were commented on by the test persons. The experiment showed a content error in question 9, which could not be answered correctly due to a programming error in the immersive anatomy atlas. The question was therefore not evaluated. The following calculations therefore refer to *n* = 9 questions.

### Error rates

**Figure 4:**
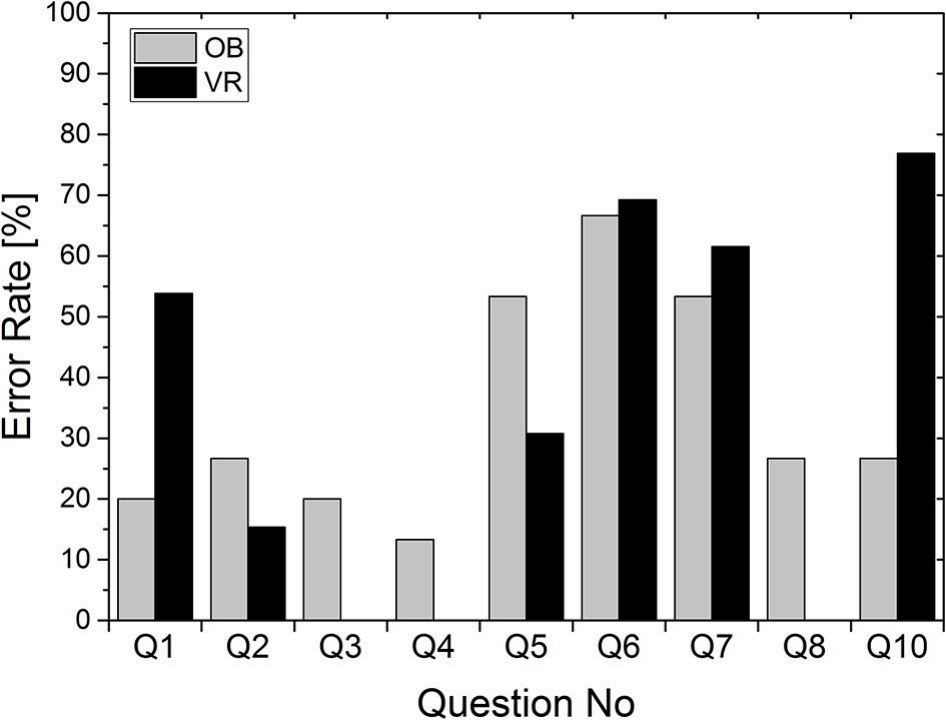
Error rate in percent depending on the question.

The three-way ANOVA showed no difference in the error rate in relation to the respective high school or the respective learning condition. The average error rate in the VR group was 34.2%, in the OB group 34.1% (*p* > 0.05). Clear but not significant differences were found between the error rates for the respective questions [*F*_(1.35)_ = 2,913; *p* = 0.076; see Figure 4). The VR error rate was at least equal to or better than the error rate of the OB group, except for questions 1 and 10.

### Processing time

**Figure 5:**
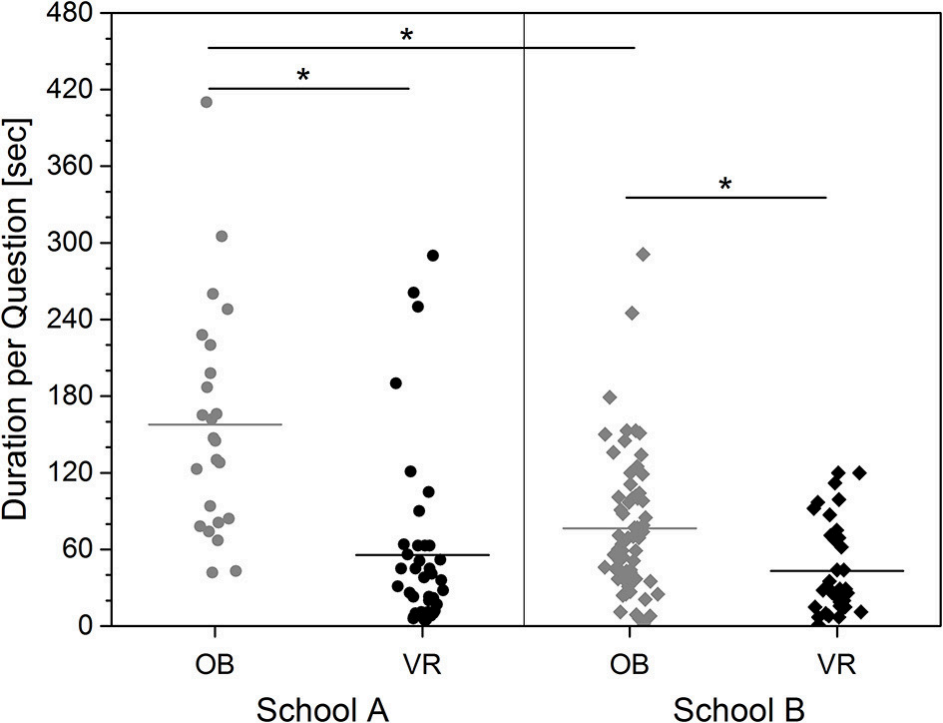
Processing time for correctly answered questions depending on school affiliation and teaching conditions. The individual measured values and the average value per group are shown.

The average processing time for all correctly processed questions over all participants was 76 s per question (range: 1–410 s; see Figure 5). The average processing time in the VR Group was 50 s per question (range: 1–290 s). The average processing time in the OB group was 98 s (range: 2–410 s) per question. Thus, the processing time for the OB group is on average twice as high as for the VR group.

The Kruskal-Wallis One Way ANOVA showed a significant difference between the four groups School A in OB and VR, and School B in OB and VR [*H*_(3)_ = 44.324; *p* < 0.001]. The average processing time in the OB group in school A was 158 s (range: 42–410 s) and in the VR group 56 s (range: 5–290; Figure 5). In school B the average processing time in the OB group was 77 s (range: 2–291 s.) and in the VR group 42 s (range: 1–120 s). The group differences between schools and teaching methods are significant, except the difference between VR in both schools (Dunn's All Pairwise Multiple Comparison: Q always > 3.1; p always < < 0.05). Specifically, the pupils in school A seem to benefit more from the immersive learning method.

### Subjective assessment of the teaching unit by students of high school B

**Figure 6:**
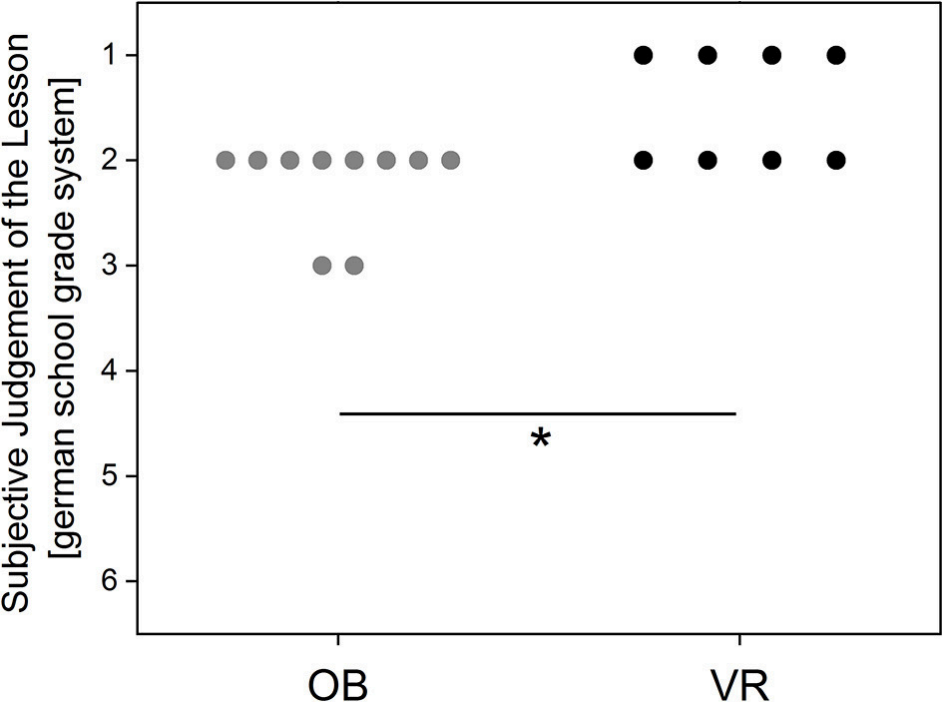
Evaluation of the teaching unit using the German school grading system (1–6; with 6 as the lowest grade). Every point represents a participant's answer.

The subjective survey on the learning methods showed a significantly better school grade for the VR learning method (Figure 6; Mann-Whitney *U*-Statistic = 16.0; T = 52; *p* = 0.012).

**Figure 7:**
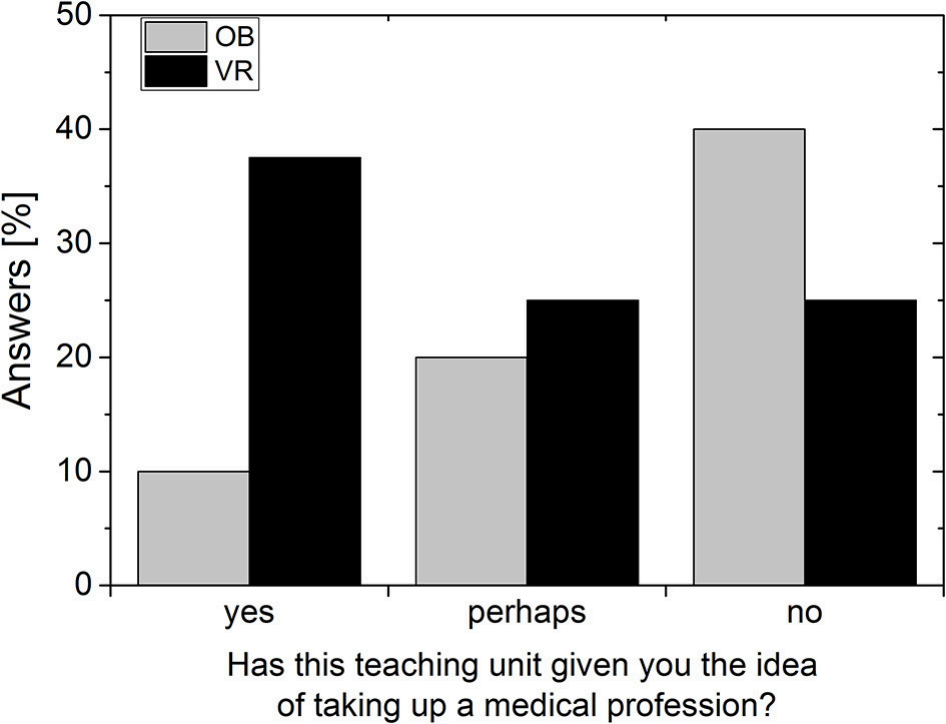
Answers to the question about the desired career. Data per group (OB or VR) in percent of students surveyed.

The immersive teaching unit seems to have additionally aroused the desire and interest in the medical field (Figure 7). In the OB Group, on the other hand, interest seems to decline. However, these results should be interpreted cautiously, because of the small number of students answering this question.

## Discussion

The present study examines the acceptance and ease of use of a virtual anatomy atlas in a group of young people without specific prior anatomical knowledge in order to avoid bias. The fact that the results are not influenced by previous knowledge is shown by the almost equal error rates in VR and OB groups of 34.2 and 34.1% respectively. However, it seems as if most questions were easier to answer in the VR environment, since VR showed higher error rates only in two questions. A reason for the higher error rate for VR regarding question 1 might be a simple left/right orientation problem, whereas higher error rates for question 10 indicate a lower level of detail in VR, which will be corrected in future versions. Overall, one may conclude that the amount of knowledge to be gained is comparable between both conditions, and depends on the type of question.

However, our results also seem to reflect the learning environment of the students. In the immersive condition, students of both schools took approximately the same time to complete the tasks. With the OB method, however, the students who were less trained in text comprehension needed significantly longer to find a solution than the more trained group. This indicates that the intuitive interaction and the playful approach in the VR condition is more accessible to everyone than the more traditional learning method, for which one must acquire at least some knowledge about text interpretation. This more traditional method of knowledge acquisition and processing seems more complex and it seems as if it has to be specifically trained. In addition, our observations during the study support our considerations of a potentially increased intrinsic motivation through playful learning using the VR approach.

OB and VR students in high school B, who are all very well-trained in text comprehension, show little difference in answering duration. However, the conventional teaching medium is not only rated worse, but beyond that, it reduces interest in the medical field. In comparison, the VR group showed twice as much potential interest in taking up a medical profession. These results are comparable with the findings of Fairén et al. (21), who showed that satisfaction of students' expectations was high in a VR anatomic course. In the randomized groups, our pilot study not only shows higher satisfaction with the VR teaching method, it also increased the interest to take up a medical profession.

In any case, the constructivist type of learning enabled by the VR anatomy atlas seems to lead to a faster solution, since the participants of the VR group found the right solution in a 50% shorter time. It is conceivable that the interactive and thus constructivist learning methodology of the immersive anatomy atlas has made it possible to better understand the information sought through active actions and thus to solve it more quickly. Constructivist learning by definition means that through the interaction of cognitive performance and simultaneous physical activity new and unknown topics can be grasped and classified more quickly.

This increase in learning efficiency and the fun of learning when using immersive digital media has led to the rapid further development of VR and AR technology in recent years as well as the development of various tools in the medical field, for example for learning anatomy or various (surgical) procedures (32–38). However, this development is now also leading to increased discussions about whether cadaver training is still up-to-date (39–43). With the current state of the art, from our point of view cadaver training is still irreplaceable insofar as it offers haptic feedback that cannot currently be produced with VR simulations. In addition, VR and AR systems are currently not designed for several simultaneous users, an important prerequisite for an educational exchange between pupils or between teacher and pupils. Thus, integration of haptic feedback as well as possibilities for several users in one OP simulator are current important research topics.

However, our study also shows that compared to the open-book method, the immersive anatomy atlas can currently already improve the learning effect for anatomical structures. Thus, with the help of the immersive anatomy atlas it was obviously easier for the participants to actively perform a targeted action according to the question, which then quickly led to a correct answer in over 60% of the questions, even without prior anatomical knowledge. With the conventional book method, orientation difficulties and/or the manual effort in using the topographical anatomy atlas in general seem to lead to a significantly longer response time. As shown by the fact that in the OB condition students from school B trained in text analysis were significantly faster than the untrained students from school A, but they still needed twice as long as the VR group from the same school to find the right solution.

Unclear in our study is the retention rate of the acquired knowledge. Further limitations of this study are the use of a non-scientifically validated questionnaire and not using a standardized intelligence test. For medical students, the benefit of the VR atlas could be, for example, that they are supported by the interactivity in memorizing names of and positional relationships between anatomical structures. Further studies with medical students should therefore be developed and carried out together with specialized learning theorists, psychologists, and university didactics to develop informative tasks more geared toward retention and spatial relations between anatomical structures.

## Conclusions

The comparative study of the usability of a VR anatomy atlas in high school students without previous anatomical knowledge shows not only that correct answers might be found 50% faster with the help of the digital medium. It also shows a higher acceptance of the learning unit. The effect is particularly clear for students learning in a “smart” learning environment. Students specially trained in text analysis are comparatively good in using a more traditional way to access knowledge, but even they profit significantly from the digital teaching medium. Further scientific interdisciplinary studies should follow this pilot study to formulate and validate the basis of a digital-based constructivist learning theory in medical studies.

## Author contributions

DW and FW developed the study design. FW carried out the measurements at school A. VU carried out the measurement at school B together with two colleagues and was mainly responsible for the analyses of the data and the creation of the figures. DW and VU wrote the manuscript. MK developed the anatomy atlas with the help of several University of Bremen students and described the technical aspects of the anatomy atlas in the manuscript. GZ supervised the Bremen students and MK. All authors have revised the manuscript.

### Conflict of interest statement

The authors declare that the research was conducted in the absence of any commercial or financial relationships that could be construed as a potential conflict of interest.

## Supplementary material

The Supplementary Material for this article can be found online at: https://youtu.be/gGsLkDfxqLc and https://youtu.be/JY50Wjh-olw

## References

[1] Malone TW editor. What makes things fun to learn? Heuristics for designing instructional computer games. In: Proceedings of the 3rd ACM SIGSMALL Symposium and the First SIGPC Symposium on Small Systems (Palo Alto, CA: ACM) 1980.

[2] Malone TW Toward a theory of intrinsically motivating instruction. Cogn Sci. (1981) 5:333–69.

[3] Lepper M “Intrinsic motivation and instructional effectiveness in computer-based education” in: Aptitude, Learning, and Instruction: III. Conative and Affective Process Analyses. Erlbaum: Hillsdale (1987). p. 255–96.

[4] Hoblitz A Spielend Lernen im Flow: Die Motivationale Wirkung von Serious Games im Schulunterricht. Heidelberg; Berlin; Germany: Springer-Verlag (2015).

[5] David M Sande C Serious Games: Games That Educate, Train, and Inform. Boston, MA: Course technology, Cengage Learning (2006).

[6] Egenfeldt-Nielsen S Overview of research on the educational use of video games. Nord J Digit Liter. (2006) 1:184–214. Available online at: https://www.idunn.no/dk/2006/03/overview_of_research_on_the_educationaluseof_video_games?mode=pr

[7] Egenfeldt-Nielsen S Beyond Edutainment: Exploring the Educational Potential of Computer Games. Copenhagen: Lulu; Game-Research.com (2011).

[8] Kirriemuir J McFarlane A Literature review in games and learning. In: NESTA Futurlab Series. Bristol: NESTA Furturelab (2004). Available online at: www.nestafuturelab.org/research/reviews/08_01.htm (Accessed Accessed: November 22, 2018)

[9] McFarlane A Sparrowhawk A Heald Y Report on the Educational Use of Games. TEEM (Teachers evaluating educational multimedia), Cambridge (2002).

[10] Mitchell A Savill-Smith C The Use of Computer and Video Games for Learning: A Review of the Literature. London: Learning and Skills Development Agency (2004). Available online at: http://dera.ioe.ac.uk/5270/7/041529_Redacted.pdf (Last Accessed November 22, 2018).

[11] Williamson B Sandford R (editors). Playful pedagogies: Cultural and curricular approaches to game-based learning in the school classroom. In: Williamson B Sandford R. editors. Handbook of Research on Improving Learning and Motivation Through Educational Games: Multidisciplinary Approaches. IGI Global: Hershey (2011). p. 846–59.

[12] Boyle EA Hainey T Connolly TM Gray G Earp J Ott M An update to the systematic literature review of empirical evidence of the impacts and outcomes of computer games and serious games. Comput Educ. (2016) 94:178–92. 10.1016/j.compedu.2015.11.003

[13] Hainey T Connolly TM Boyle EA Wilson A Razak A A systematic literature review of games-based learning empirical evidence in primary education. Comput Educ. (2016) 102:202–23. 10.1016/j.compedu.2016.09.001

[14] Merchant Z Goetz ET Cifuentes L Keeney-Kennicutt W Davis TJ Effectiveness of virtual reality-based instruction on students' learning outcomes in K-12 and higher education: a meta-analysis. Comput Educ. (2014) 70:29–40. 10.1016/j.compedu.2013.07.033

[15] The Practice of Constructivism in Science Education Tobin K editor. New York, NY: Routledge (1995).

[16] Siemens G Connectivism: A learning theory for the digital age. Int J Instr Tech Distance Learn. (2005) 2. Available online at: http://www.itdl.org/Journal/Jan_05/article01.htm (Last Accessed November 22, 2018).

[17] Fosnot CT Perry RS Constructivism: A psychological theory of learning. In: Eurographics 2017: Education Papers Lyon: Eurographics (2017).

[18] Neubert S Reich K Voß R Lernen als konstruktiver Prozess. In: Hug T editor. Einführung in Das Wissenschaftliche Arbeiten Wie kommt Wissenschaft zu Wissen? Vol 1. Baltmannsweiler: Schneider Verlag Hohengehren (2001). p. 253–65.

[19] Seymour NE. VR to OR: a review of the evidence that virtual reality simulation improves operating room performance. World J Surg. (2008) 32:182–8. 10.1007/s00268-007-9307-9 18060453

[20] McGaghie WC Issenberg SB Petrusa ER Scalese RJ. A critical review of simulation-based medical education research: 2003–2009. Med Educ. (2010) 44:50–63. 10.1111/j.1365-2923.2009.03547.x 20078756

[21] Fairén González M Farrés M Moyes Ardiaca J Insa E editors. Virtual Reality to teach anatomy. In: Eurographics 2017: Education Papers. Lyon: European Association for Computer Graphics (Eurographics) (2017).

[22] Rauber A Kopsch F Anatomie des Menschen Bd. 1-4. Stuttgart; New York, NY: Thieme (1987).

[23] Kurashima Y Hirano S. Systematic review of the implementation of simulation training in surgical residency curriculum. Surg Today (2017) 47:777–82. 10.1007/s00595-016-1455-9 28004190

[24] Bosse HM Mohr J Buss B Krautter M Weyrich P Herzog W. The benefit of repetitive skills training and frequency of expert feedback in the early acquisition of procedural skills. BMC Med Educ. (2015)15:22. 10.1186/s12909-015-0286-5 25889459 PMC4339240

[25] Carlsen CG Lindorff-Larsen K Funch-Jensen P Lund L Konge L Charles P. Module based training improves and sustains surgical skills: a randomised controlled trial. Hernia (2015) 19:755–63. 10.1007/s10029-015-1357-6 25731946

[26] Sonnadara RR Van Vliet A Safir O Alman B Ferguson P Kraemer W. Orthopedic boot camp: examining the effectiveness of an intensive surgical skills course. Surgery (2011) 149:745–9. 10.1016/j.surg.2010.11.011 21236456

[27] Palter VN Orzech N Aggarwal R Okrainec A Grantcharov TP. Resident perceptions of advanced laparoscopic skills training. Surg Endosc. (2010) 24:2830–4. 10.1007/s00464-010-1058-2 20428895

[28] Chang L Petros J Hess DT Rotondi C Babineau TJ. Integrating simulation into a surgical residency program. Surg Endosc. (2007) 21:418–21. 10.1007/s00464-006-9051-5 17180282

[29] Stefanidis D Heniford BT. The formula for a successful laparoscopic skills curriculum. Arch Surg. (2009) 144:77–82. 10.1001/archsurg.2008.528 19153329

[30] Weber EMW Schemata der Leitungsbahnen des Menschen. Heidelberg; Berlin: Springer (1999).

[31] Paulsen F Waschke J Sobotta, Atlas der Anatomie des Menschen Bd 1-3. München; Jena: Urban & Fischer Verlag; Elsevier Health Sciences (2011).

[32] Alaker M Wynn GR Arulampalam T. Virtual reality training in laparoscopic surgery: A systematic review & meta-analysis. Int J Surg. (2016) 29:85–94. 10.1016/j.ijsu.2016.03.034 26992652

[33] Yiannakopoulou E Nikiteas N Perrea D Tsigris C. Virtual reality simulators and training in laparoscopic surgery. Int J Surg. (2015) 13:60–4. 10.1016/j.ijsu.2014.11.014 25463761

[34] Huber T Paschold M Hansen C Wunderling T Lang H Kneist W. New dimensions in surgical training: immersive virtual reality laparoscopic simulation exhilarates surgical staff. Surg Endosc. (2017) 31:4472–7. 10.1007/s00464-017-5500-6 28378077

[35] Jain N Youngblood P Hasel M Srivastava S. An augmented reality tool for learning spatial anatomy on mobile devices. Clin Anat. (2017) 30:736–41. 10.1002/ca.22943 28631297

[36] Bernardo A. Virtual reality and simulation in neurosurgical training. World Neurosurg. (2017) 106:1015–29. 10.1016/j.wneu.2017.06.140 28985656

[37] Ellington DR Shum PC Dennis EA Willis HL Szychowski JM Richter HE. Female pelvic floor immersive simulation: a randomized trial to test the effectiveness of a virtual reality anatomic model on resident knowledge of female pelvic anatomy. J Minim Invas Gynecol. (2018). 10.1016/j.jmig.2018.09.003. [Epub ahead of print]. 30218709

[38] Pulijala Y Ma M Pears M Peebles D Ayoub A. Effectiveness of immersive virtual reality in surgical training—A randomized control trial. J Oral Maxillofac Surg. (2018) 76:1065–72. 10.1016/j.joms.2017.10.002 29104028

[39] Konschake M Brenner E “Mors auxilium vitae”—Causes of death of body donors in an Austrian anatomical department. Ann Anat. (2014) 196:387–93. 10.1016/j.aanat.2014.07.002 25107479

[40] McMenamin P McLachlan J Wilson A McBride J Pickering J Evans D. Do we really need cadavers anymore to learn anatomy in undergraduate medicine? Med Teach. (2018). 10.1080/0142159X.2018.1485884. [Epub ahead of print]. 30265177

[41] Lippert H Medizinstudium: Sind Präparierübungen an der Leiche noch zeitgemäß? Dtsch Arztebl. (2012) 109:A-1758/B-422/C-140. Available online at: https://www.aerzteblatt.de/archiv/128775/Medizinstudium-Sind-Praeparieruebungen-an-der-Leiche-noch-zeitgemaess

[42] Ferrada P Anand RJ Amendola M Kaplan B. Cadaver laboratory as a useful tool for resident training. Am Surg. (2014) 80:408. 24887675

[43] Sharma M Macafee D Horgan AF. Basic laparoscopic skills training using fresh frozen cadaver: a randomized controlled trial. Am J Surg. (2013) 206:23–31. 10.1016/j.amjsurg.2012.10.037 23623462

